# 

**Published:** 1854-02

**Authors:** 


### The Western Journal

OF

### MEDICINE AND SURGERY.

### NEW SERIES.

The Old Series of this Periodical, consisting of Twenty-eight volumes, was brought to an abrupt termination, by the destruction of the "Louisville Journal" office, by fire, in November, 1853. Arrangements have been made with Mr. J. F. Brennan, Publisher, for its continuation, under more favorable auspices, and in a much improved form. It has been considered prudent to reduce the price of the work to subscribers from $5 to $3 per annum, payment to be invariably made in advance, and each subscription to commence with the present number,—a supply of which has been printed sufficient to satisfy all presumable demand. The Editor guarantees to procure of his colleagues, in the University of Louisville, articles for each number, which, with his own productions and selections from the leading Medical publications of Europe and America, will, he flatters himself, afford the subscribers and readers of the Western Journal, a monthly periodical readable and instructive, and well worth the price at which the publisher supplies it.

The arrangement made with the new publisher of this work does not demand of him responsibility for the failure, in any way, of the old issue. This is distinctly understood. Such responsibility will be met by the late publisher, or his assigns; and communications upon that subject should be so directed, to receive attention.

The present issue of this work will consist of Eighty pages a month. With the closing or December number, a title-page and copious Index will be supplied; and the publisher will be prepared to bind the work in a handsome uniform manner, in embossed muslin, at a small cost.

☞ All communications, containing subscription or any other business matter connected with the publishing department, should be addressed to

J. F. BRENNAN, Box 126, Louisville, Ky.

and all pertaining to the editorial department, to the Editor, L. P. YANDELL, M. D., Louisville, Ky.

☞ Subscription, Three Dollars per annum. No name entered until the money is received.

